# Effects of Marital Status on Cochlear Implant Outcomes

**DOI:** 10.1002/lary.70178

**Published:** 2025-09-30

**Authors:** Barak M. Spector, John P. Doran, Katelyn A. Berg, Aaron C. Moberly, Lijun Song, Terrin N. Tamati

**Affiliations:** ^1^ Department of Otolaryngology–Head and Neck Surgery Vanderbilt University Medical Center Nashville TN USA; ^2^ Department of Sociology, Center for Medicine, Health, and Society Vanderbilt University Nashville TN USA

**Keywords:** Cochlear implant, marital status, speech recognition

## Abstract

**Objective:**

To examine the association between marital status and post‐CI speech recognition and hearing‐related QoL in adult CI users, and to explore how this relationship interacts with key sociodemographic factors, including sex, employment, and residential location, and device usage (datalogging).

**Methods:**

Retrospective cohort analysis of prospectively collected data (2018–2024) from 604 postlingually deafened adults undergoing unilateral cochlear implantation. Outcomes were speech recognition (AzBio, CNC) and hearing‐related QoL (CIQOL), collected at 6 or 12 months post‐CI.

**Results:**

Unmarried users had significantly poorer speech recognition outcomes (CNC [95% CI −15.2 to −5.5], AzBio quiet (AzBioQ) [95% CI −15.2 to −2.8]) compared to married users when controlling for key clinical covariates. No significant differences were observed in CIQOL scores. There were positive nonsignificant interactions between marital status and sex (*β*, 9.33, [95% CI −3.44 to 22.12]), employment status (*β*, 8.52, [95% CI −6.47–23.51]), and residential location (*β*, 12.96, [95% CI −0.54–26.46]) on AzBioQ. The benefits of marriage were stronger among women, full‐time employed individuals, and rural residents than among men, those not in full‐time employment, and urban residents. Marital status also significantly interacted with device usage (*β*, 1.70, [95% CI 0.11–3.28]). The relationship between device usage and AzBioQ was stronger in married individuals, suggesting a greater protective effect of marriage in individuals with higher device usage.

**Conclusion:**

Marital status predicts CI speech recognition outcomes through both its main effect and its interaction with three additional sociodemographic factors and device usage. Recognizing this significant impact can help guide clinical counseling and inform the importance of social relationships.

**Level of Evidence:**

**3**.

## Graphical abstract

Marital status was evaluated as a predictor of post‐CI speech recognition and hearing‐related QoL in 604 adults undergoing cochlear implantation. Unmarried individuals had significantly poorer speech recognition outcomes, while QoL scores did not differ; benefits of marriage were most evident among women, employed individuals, and rural residents, and were amplified with greater device usage. These findings highlight marriage as an important social factor influencing CI outcomes and emphasize the role of social relationships in clinical counseling.

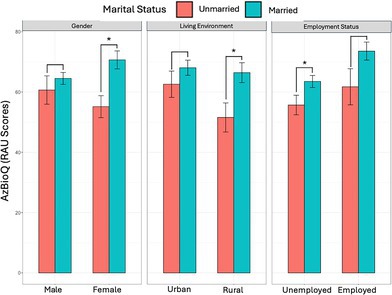

## 1 Introduction

Cochlear implants (CIs) are an effective intervention for adults with moderate‐to‐severe hearing loss (HL), providing a sense of hearing and improved speech communication [1]. However, speech recognition outcomes vary widely among adult CI users [2, 3, 4, 5, 6, 7]. While several demographic, audiological, and surgical factors contributing to CI outcomes have been identified [4, 5, 8, 9], many of these factors are not malleable and remain outside patient or clinician control. In contrast, social factors may represent modifiable influences on CI outcomes. The current study focused specifically on marital or partnered status as one such social factor that was hypothesized to impact CI outcomes.

Broadly, the perspective on social networks (i.e., structures of relationships linking social actors) offers a valuable lens for identifying modifiable social factors that may influence CI success [10]. Social integration theory states that involvement in social relationships protects health through multiple mechanisms, particularly social support (aid or assistance from network members) and social control (the influence of network members on individuals' attitudes and behaviors) [11, 12, 13]. In the context of CIs, spouses or cohabiting partners serve as consistent communication partners (CPs) who can provide multiple forms of social support (e.g., emotional, informational, instrumental, and appraisal) that facilitate device use, rehabilitation, and communication success. In addition, spouses or partners can exert social control by encouraging continued device use and adherence to rehabilitation practices.

Marital status is a well‐established determinant of overall health and well‐being, with married individuals generally experiencing better physical, cognitive, and emotional health and well‐being compared to their unmarried counterparts [14]. Additionally, the health impact of marital status varies by sociodemographic factors. Notably, the benefits of marriage are typically more pronounced among men than women [15, 16]. Furthermore, the health advantages of marriage are less evident among individuals who are fully employed compared to those who are not [17].

Despite this large body of evidence, however, little research has examined the role of marital status in the context of CI outcomes. For example, McRackan et al. found no significant evidence for the impact of marital status on post‐CI hearing‐related quality of life (QoL), as measured by the Cochlear Implant Quality of Life (CIQOL) questionnaire [18]. However, their analysis focused solely on self‐reported QoL and did not examine potential interactions between marital status and other factors. In contrast, Tang et al. reported that CI users who lived with others scored higher on sentence recognition tasks than those who lived alone [19]. Yet, their study was constrained by a modest sample size (*N* = 76) and also did not account for the potential confounding or interaction effects of other factors.

While not examining CI outcomes, some studies highlight the positive role of spouses in hearing care. Both Van Leeuwen et al. and Engdahl and Aarhus found that marital status was a significant predictor of hearing aid uptake, with married individuals being more likely to adopt hearing aids compared to their unmarried counterparts [20, 21]. Similarly, Tolisano et al. observed that married individuals were more likely to pursue CIs than their unmarried counterparts [22]. Taken together, these gaps highlight the need for research examining the role of marital status in CI outcomes. Therefore, the primary objective of this study was to investigate the association between marital status and CI outcomes, while considering potential interactions with four factors: three sociodemographic factors (sex, employment status, and residential location) and a CI usage factor (measured via datalogging). We examined the impact of marital status on speech recognition and hearing‐related QoL measured at 6 and 12 months post‐CI. Applying social integration theory, we hypothesized a positive association between marital status and CI outcomes.

We also explored whether sex, employment status, and residential location (urban versus rural zip code) modify the relationship between marital status and CI outcomes. These potential moderators were selected based on prior evidence that the salience and impact of romantic relationships differ across social contexts. Although men often experience greater health benefits of marriage than women, women may derive more benefits from romantic partnerships in the context of CI use due to their high levels of device engagement. Women may use their devices more actively than men to maintain social bonds, in line with gendered norms around relational, caregiving, emotional, and communicative responsibilities [23, 24, 25]. Similarly, individuals with full‐time jobs may face more sustained communication demands and engage in more diverse listening and communicative experiences, both of which may encourage greater device usage and enhance the role of romantic partners. Alternatively, employment may reduce the relative benefit of marital status since individuals with full‐time jobs would already have access to rich communicative opportunities. In rural areas, where individuals often face reduced access to social resources and interaction opportunities due to geographic and infrastructural isolation, romantic partners may become especially central as sources of social support and control [26]. We hypothesized that the positive impact of marital status on CI outcomes would be stronger among women, full‐time employed individuals, and rural residents than among men, those not in full‐time employment, and urban residents.

Finally, the influence of social relationships may differ when individuals are more actively engaged in listening behaviors, such as device usage, creating opportunities for reinforcement, monitoring, and mutual communication. Prior research has shown that device usage hours (measured via datalogging) are positively associated with CI outcomes [27, 28]. Higher levels of CI use may amplify the effects of social support and control from spouses or partners, making romantic relationships more impactful. Thus, we also expected that married CI users would benefit more from higher device use than unmarried CI users.

## 2 Methods

### 2.1 General Procedures

A retrospective review of clinical data identified adults who underwent cochlear implantation from 2018 to 2024. All data were collected prospectively within the clinical practice of a tertiary care adult CI center and reviewed retrospectively. The study was approved as exempt under IRB #240876.

### 2.2 Participants

From our clinical CI database (1197 total patients), 604 patients with pertinent variables were identified. These variables included clinical predictors (e.g., age at implantation, low frequency PTA, duration of deafness, daily CI usage, and pre‐CI speech recognition), sociodemographic factors (sex, marital status, employment status, and residential location), and outcome measures (post‐CI speech recognition and hearing‐related QoL). Patients with bilateral implantation or retrocochlear pathology were excluded. Duration of deafness was defined as the period since the patient first experienced significant difficulty with hearing aids or since a notable change in hearing occurred (e.g., sudden sensorineural HL).

Post‐CI speech recognition scores and QoL were typically collected clinically at 6, and/or 12 months post‐activation. All CI users were post‐lingually deafened adults with bilateral HL qualifying for cochlear implantation, defined as unaided PTA > 60 dB HL and best‐aided CNC words (CNC) < 60% in both ears, and officially demonstrating CI candidacy with best‐aided AzBio sentence scores in quiet (AzBioQ) or in noise < 60%.

### 2.3 Sociodemographic Factors

Biologic sex (female = 1, male = 0) was collected as reported in the medical chart. Marital status (married = 1, unmarried = 0) and employment status (employed full time = 1, unemployed or retired = 0) were determined based on previous encounter notes with medical professionals. These data were most often obtained from CI candidacy evaluation notes. When not available, we collected them from clinical encounter notes dated between the time of CI evaluation and the date of surgery. This approach ensured that marital and employment status reflected the period closest to cochlear implantation, minimizing potential misclassification. Participants with specified domestic partnerships were included in the married group. Participants with unknown marital or partnered status or with unknown or part‐time jobs were excluded from our analysis. Residential location (rural = 1, urban = 0) was determined by converting patient zip codes to rural–urban commuting area (RUCA) codes using publicly available classification data from the US Department of Agriculture (USDA).

### 2.4 CI Device Usage

Daily CI usage was defined as the mean number of hours of processor use per day, as documented in clinical reports via datalogging. These definitions of duration of deafness and CI device usage were consistent with the methodology described by Defreese et al. [28].

### 2.5 Hearing‐Related QoL

Hearing‐related QoL was assessed using the CIQOL‐10 [29], which provides a single, comprehensive measure reflecting the broad impact of cochlear implantation on functional abilities and overall well‐being. Scores range from 0 (indicating the poorest QoL) to 100 (indicating the highest QoL), such that higher scores represent better hearing‐related QoL.

### 2.6 Data Analyses

All continuous variables were initially assessed for normality using skewness and kurtosis values, with thresholds of > 1 and > 3, respectively, indicating substantial deviation from normality. Several variables, including the duration of deafness, exceeded these thresholds, prompting the use of nonparametric tests (e.g., Wilcoxon Rank–Sum) for group comparisons.

Given the prior research suggesting a plateau in speech recognition outcomes at around 6 months of CI use [30], post‐CI speech‐recognition and CIQOL scores collected at 6 or 12 months post‐CI (whichever score was higher) were collapsed and treated as post‐CI outcomes for analyses.

For regression analyses, continuous predictors were retained in their original form to preserve interpretability, except for speech recognition scores, which were transformed from percent correct to rationalized arcsine units (RAU) to reduce the influence of floor and ceiling effects [31]. Assumptions of linearity and homoscedasticity were confirmed via residual plots, and results were consistent when reanalyzed using log‐transformed versions of skewed variables (e.g., duration of deafness, CI device usage).

To examine the main effects of marital status, Wilcoxon Rank–Sum tests were first used to compare post‐CI speech recognition and QoL outcomes between married and unmarried individuals. For outcome measures showing significant group differences, multivariable linear regressions were conducted, including marital status and clinical covariates (age at implantation, pre‐CI LFPTA, duration of deafness, pre‐CI speech recognition).

To investigate potential interaction effects, additional Wilcoxon Rank–Sum tests were performed to explore differences in speech recognition across key sociodemographic subgroups (sex, employment status, and residential location) within marital status groups. *p*‐Values were adjusted using the false discovery rate (FDR) correction, with *α* = 0.05. Interaction effects were further examined via four supplemental regression models, each including marital status and one interaction term (e.g., Marital status × sex, employment status, residential location, and CI device usage). These models reported unstandardized beta coefficients and 95% confidence intervals for the interaction terms, along with adjusted *R* ^2^ values. Statistical significance was defined as *p* < 0.05.

## 3 Results

Participants ranged in age from 20 to 84 years (mean = 60.2; SD = 15.5). None of the participants had prior CI surgery or a history of CI revision. The study sample's demographics are detailed in Table 1.

**TABLE 1:** Demographic characteristics of the study sample.

Demographics	
Characteristics	No. (%)
Marital status	
Married/domestic partnership	418 (69.2%)
Not married/no domestic partnership	186 (30.8%)
Sex	
Male	332 (55.0%)
Female	272 (45.0%)
Residential location	
Urban	260 (43.0%)
Rural	163 (57.0%)
Employment status	
Employed	201 (33.3%)
Not employed/retired	403 (66.7%)

### 3.1 Does Marital Status Influence Cochlear Implant Outcomes?

#### 3.1.1 Speech Recognition and QoL

Wilcoxon Rank–Sum tests were performed to compare CI outcomes between married and unmarried participants (see Table 2). Married individuals showed significantly better outcomes for CNC and AzBioQ (*p* ≤ 0.004). No significant difference between married and unmarried individuals was observed for AzBioN and CIQOL.

**TABLE 2:** Mean post‐CI outcomes for married and unmarried individuals.

	Married (*N*)	Unmarried (*N*)	95% CI
Post‐CI outcomes (CI ear)			
CNC words (%)	54.10 (22.86)	44.86 (23.66)	**−15.2 to −5.5**
AzBio sentence, quiet (%)	66.08 (26.61)	57.60 (29.02)	**−15.2 to −2.8**
AzBio sentence, noise (%)	29.54 (21.54)	28.82 (18.60)	−6.2 to 7.3
Post‐CI PROMS			
CIQOL‐10 global	51.50 (9.22)	50.10 (13.80)	−7.8 to 1.7

*Note*: Wilcoxon Rank–Sum tests were performed between each group. 95% confidence intervals are reported, reflecting the difference between groups referenced to the married group; significant *p*‐values after FDR‐correction (< 0.05) are bolded.

Based on the significant results that emerged from Wilcoxon‐Sum Tests, we conducted linear regression analyses evaluating the effect of marital status on CNC and AzBioQ, accounting for key clinical predictor variables (see Table 3). Marital status significantly predicted better outcomes: married individuals scored an average of 7.67% higher on both CNC and AzBioQ tasks.

**TABLE 3:** Multivariable regression analyses evaluating the effect of marital status on post‐CI CNC word and AzBio sentences in quiet, accounting for key clinical predictor covariates.

Measurement	*β* (95% CI)	*β* (95% CI)
	CNC (RAU)	AzBioQ (RAU)
*R* ^2^	0.24	0.30
Variable		
Intercept	**46.34 (31.38–61.31)**	**72.99 (52.89–93.10)**
Marital status	**7.67 (2.59–12.76)**	**7.67 (1.37–13.98)**
Age at implantation (years)	**−0.23 (−0.38 to −0.01)**	**−0.59 (−0.79 to −0.40)**
Pre‐CI LFPTA	**−0.12 (−0.23 to −0.01)**	−0.09 (−0.22 to 0.46)
Duration of deafness (years)	−0.27 (−0.59 to 0.05)	**−0.39 (−0.79 to 0.00)**
Pre‐CI speech recognition score (RAU)	0.**15 (0.04–0.27)**	0.**16 (0.05–0.28)**
CI device usage (h)	**2.22 (1.59–2.86)**	**3.05 (2.26–3.85)**

*Note*: Significant coefficients (*p* < 0.05) are bolded.

Other significant clinical variable predictors include **age at implantation, duration of deafness (in AzBioQ only), CI device usage, and pre‐CI speech recognition scores**. Age at implantation and duration of deafness were negatively associated with all speech recognition measures, while greater CI device usage and better preoperative speech recognition scores were associated with improved outcomes. Pre‐CI LFPTA was negatively associated with CNC, but not AzBioQ.

### 3.2 Does Marital Status Interact With Sex, Employment Status, and Residential Location?

For the remainder of the analyses, we focused on AzBio sentences, which were designed to more realistically represent everyday life speech patterns and content relative to CNC words [32]. This decision is further supported by the strong correlation (*r* = 0.83, *p* < 0.001) between AzBioQ and CNC scores, indicating redundancy in analyzing both measures.

We evaluated potential interactions between marital status and the other three sociodemographic variables by adding each interaction term separately to the regression model predicting AzBioQ outcomes (Table 4).

**TABLE 4:** Summary of additional regression models examining interactions between marital status and sociodemographic factors/CI device usage.

Interaction term	*R* ^2^	*β* (95% CI)
Marital status × sex	0.30	9.33 (−3.44 to 22.12)
Marital status × residential location	0.26	8.52 (−6.47 to 23.51)
Marital status × employment status	0.31	12.96 (−0.54 to 26.46)
Marital status × CI device usage	0.31	1.70 (0.11–3.28)

*Note*: Each *R* ^2^ of the new model, in addition to the *β* and 95% confidence interval of the interaction term.

**FIGURE 1:**
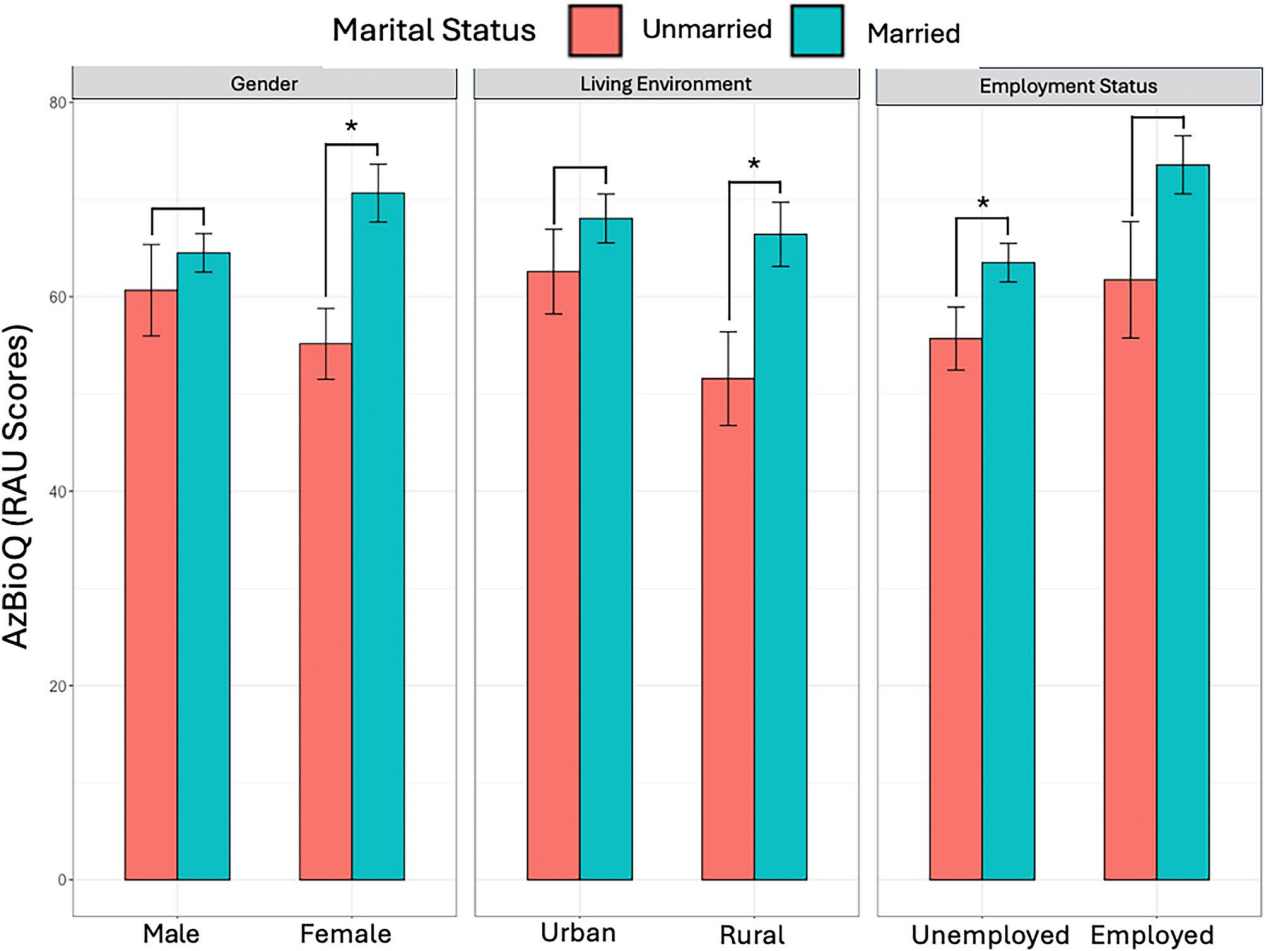
Mean AzBioQ scores comparing unmarried and married CI users across three demographic factors: sex (male vs. female), residential location (urban vs. rural), and employment status (unemployed vs. employed). Error bars represent ±1 standard error. Brackets represent individuals Wilcoxon Rank–Sum test; *Significant differences after FDR correction. [Color figure can be viewed in the online issue, which is available at www.laryngoscope.com]

Marital status positively interacted with all three sociodemographic factors, but none of the interaction terms were statistically significant (Table 4): sex (*β* = 9.33), employment status (*β* = 12.96), and residential location (*β* = 8.52). However, as Figure 1 illustrates, the patterns suggest that the benefit of being married/partnered on speech recognition outcomes is stronger among female CI users, full‐time employed users, and rural users than among male CI users, those not in full‐time employment, and urban users.

#### 3.2.1 Does Marital Status Interact With Datalogging?

**FIGURE 2:**
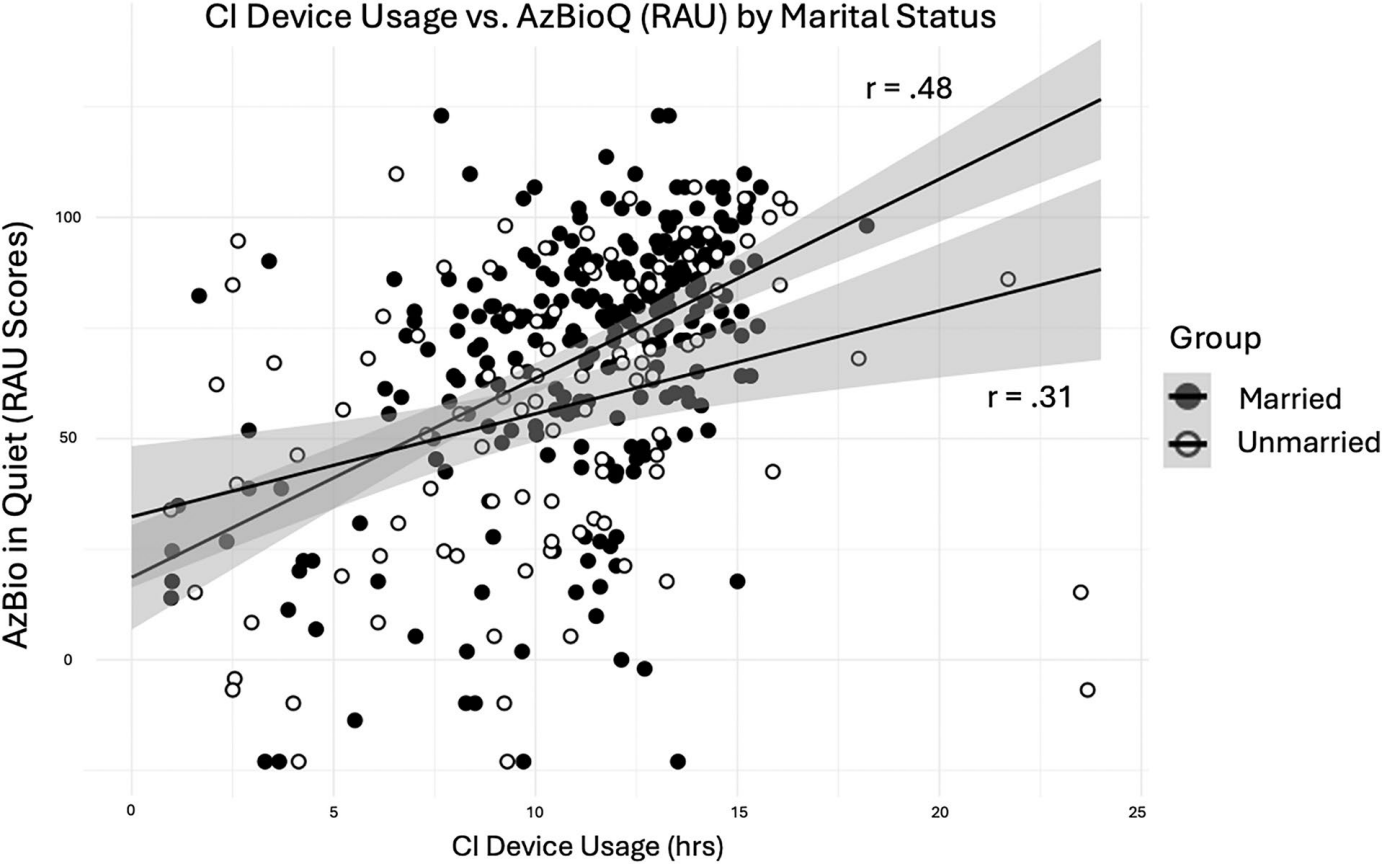
Scatterplot displaying the relationship between datalogging and AzBio scores. Plot is divided into married (*r* = 0.48) and unmarried (*r* = 0.31) participants, showing trendlines, 95% confidence interval around the trendline, and Pearson correlations (*r*).

The interaction term (Marital status × datalogging) significantly predicted AzBioQ outcomes (see Table 4, *β*, 1.70, [95% CI 0.11–3.28]). The association between marital status and speech recognition outcomes was stronger among participants with higher device usage compared to those with lower device usage. Figure 2 displays a scatterplot of datalogging and AzBioQ scores, with separate trend lines for married (*r* = 0.48) and unmarried users (*r* = 0.31). As CI device usage increased, the benefit of being married/partnered became more pronounced.

## 4 Discussion

The primary goal of this study was to investigate the main effect of marital status on CI outcomes and its interaction with three demographic variables and device usage. Our findings demonstrate that marital status is associated with speech recognition outcomes, although no effects are observed for QoL. Furthermore, the effect of marital status varies by three key sociodemographic factors and device usage, suggesting context‐specific benefits of marriage.

Consistent with social integration theory, being married was positively associated with CI speech recognition outcomes. This finding is largely consistent with the existing literature emphasizing marriage as a crucial social determinant associated with better health outcomes [14, 33, 34, 35, 36]. In the context of CIs, CI users may benefit from the social support [19, 37], encouragement of CI use and rehabilitation [38], and clear speech strategies [39] that their spouses or partners provide. Further, the presence of a consistent CP provides increased auditory engagement and potentially greater auditory input, which are likely beneficial in the rehabilitation process [27, 40].

Notably, despite the significant effect of marital status on speech recognition, we found no significant association between marital status and subjective assessments of hearing‐related QoL. This finding is consistent with previous work by McRackan et al., who also reported no significant association between marital status and CIQOL scores [18]. Although the influence of marital status on hearing‐related QoL remains uncertain, our findings underscore marital status as an important determinant of post‐CI speech recognition outcomes.

Importantly, the benefits of marriage are not uniform across subgroups. Marital status positively interacted with sex, employment status, and residential location in explaining speech recognition outcomes, although none of these interaction terms reached statistical significance. It is possible that the study was underpowered to detect multiplicative effects, or that the patterns reflect additive influences of these demographic factors rather than true interactions. Female CI users experience more pronounced speech recognition benefits from marriage than male CI users, suggesting potential sex‐specific differences in social dynamics or auditory rehabilitation approaches post‐CI. This trend is consistent with the gender norms perspective, as women are often expected to be more actively engaged in emotional, communicative, and caregiving roles within relationships [23, 24, 25].

Rural residents also appear to benefit more from being married or partnered than urban residents. Saha et al. reported that married individuals living in a rural setting have higher self‐rated health than unmarried individuals living in rural settings [41]. In the context of cochlear implantation, this may reflect the heightened importance of in‐home support and social engagement in areas with fewer audiologic resources and limited access to specialized care. As such, marriage may serve a compensatory role for rural CI users, buffering against geographic limitations.

Similarly, we observed a non‐significant trend suggesting that employment status may modify the relationship between marital status and CI outcomes. While our study was underpowered to detect small interaction effects, one possible explanation is that employed individuals experience additive social and communicative input from both the workplace and home environments, which could reinforce the benefits of marriage during auditory rehabilitation. While one alternative explanation is that employment could reduce the relative contribution of marital status by providing social and communicative support, we did not observe such a pattern. However, given the lack of statistical significance, this interpretation should be considered exploratory, and future work with larger samples is needed to clarify whether employment status meaningfully interacts with marital status in shaping CI outcomes.

Lastly, device usage significantly moderates the relationship between marital status and speech recognition outcomes. The positive association between being married or partnered and speech recognition outcomes is stronger among users with higher device usage than among those with lower device usage. Although previous research demonstrates the link between datalogging and CI speech recognition outcomes [27, 28], the specific auditory experiences that most effectively support rehabilitation remain poorly understood. Our findings suggest that the quality and context of auditory experiences (e.g., communication with a spouse or regular CP) may be critical for rehabilitation. Further research is warranted to clarify this link and examine other demographic and social factors influencing auditory experiences.

To our knowledge, this is the first study to demonstrate that social relationships, particularly romantic partnerships, shape CI speech recognition outcomes, while also controlling for key sociodemographic and audiologic factors. However, some limitations should be acknowledged. First, our retrospective data do not contain information on other romantic relationship factors such as marital quality or non‐marital partnerships. Second, employment status and residential location are derived from existing records, which may not accurately reflect participants' current work situations or residential environments. Although marital and employment status were obtained from CI candidacy notes or encounter notes dated between CI evaluation and surgery, it is possible that these statuses may have changed during that interval. However, by restricting documentation to this narrow time frame and excluding cases with unknown or ambiguous information, we believe the potential for misclassification was minimized. Third, we also did not control for education level or socioeconomic status, which may be potential confounding variables. Fourth, while datalogging measures were available, more granular variables such as hours spent in noise—which may be more impactful than total listening hours—were not captured in our dataset. Future studies incorporating these metrics will be critical to further contextualize our findings. Finally, future prospective studies should go beyond romantic relationships and incorporate broader social network measures to examine how various types of social relationships influence CI outcomes.

## 5 Conclusions

This study represents the first effort to examine the main and interaction effects of marital status on adult CI outcomes. Findings underscore the importance of network determinants and suggest that leveraging social relationships, particularly romantic partnerships, could enhance clinical speech recognition outcomes. Recognizing the importance of social relationships in rehabilitation programs may guide future patient counseling and targeted interventions, ultimately improving communication outcomes among adult CI users.

## Conflicts of Interest

Aaron C. Moberly serves as CMO and on the Board of Directors for Otologic Technologies and as a paid consultant for Amgen.

## Data Availability

The data that support the findings of this study are available from the corresponding author upon reasonable request.
